# KLF9 and EPYC acting as feature genes for osteoarthritis and their association with immune infiltration

**DOI:** 10.1186/s13018-022-03247-6

**Published:** 2022-07-28

**Authors:** Jiayin Zhang, Shengjie Zhang, Yu Zhou, Yuan Qu, Tingting Hou, Wanbao Ge, Shanyong Zhang

**Affiliations:** 1 Orthopedics, The Second Hospital of Jilin University, No. 218, Ziqiang Street, Nanguan District, Changchun, 130000 Jilin China; 2 Orthopedics, Changchun University of Chinese Medicine, No.1478, Gongnong Road, Chaoyang District, Changchun, 130117 Jilin China

**Keywords:** Osteoarthritis, KLF9, EPYC, Machine learning, Bioinformatics, Immune infiltration

## Abstract

**Background:**

Osteoarthritis, a common degenerative disease of articular cartilage, is characterized by degeneration of articular cartilage, changes in subchondral bone structure, and formation of osteophytes, with main clinical manifestations including increasingly serious swelling, pain, stiffness, deformity, and mobility deficits of the knee joints. With the advent of the big data era, the processing of mass data has evolved into a hot topic and gained a solid foundation from the steadily developed and improved machine learning algorithms. Aiming to provide a reference for the diagnosis and treatment of osteoarthritis, this paper using machine learning identifies the key feature genes of osteoarthritis and explores its relationship with immune infiltration, thereby revealing its pathogenesis at the molecular level.

**Methods:**

From the GEO database, GSE55235 and GSE55457 data were derived as training sets and GSE98918 data as a validation set. Differential gene expressions of the training sets were analyzed, and the LASSO regression model and support vector machine model were established by applying machine learning algorithms. Moreover, their intersection genes were regarded as feature genes, the receiver operator characteristic (ROC) curve was drawn, and the results were verified using the validation set. In addition, the expression spectrum of osteoarthritis was analyzed by immunocyte infiltration and the co-expression correlation between feature genes and immunocytes was construed.

**Conclusion:**

EPYC and KLF9 can be viewed as feature genes for osteoarthritis. The silencing of EPYC and the overexpression of KLF9 are associated with the occurrence of osteoarthritis and immunocyte infiltration.

## Background

Osteoarthritis (OA), a chronic joint disease prevalent among middle-aged and elderly people, is featured with degenerative changes and destructive and progressive osteogenesis of articular cartilage and has long been proven to be associated with genetic factors. About 250 million people worldwide are now troubled by the disease, which usually affects multiple joints throughout the body, with the knee joints being the most common, followed by the wrist and hip joints [1]. Its etiology has not yet been identified, but many risk factors, including heredity, sex, joint injury, age, and obesity, should be taken into account [2]. As the global population ages and the number of obese people rises, joint injury is becoming increasingly common. Some scholars suggest that mechanical damage to joints plays a leading role in the occurrence and progress of OA [3], while others describe that genetic factors are more relevant [4]. OA is clinically manifested as gradual aggravated joint pain, swelling, stiffness, dysfunction, or even disability in some severe cases. Its diagnosis predominantly resorts to imaging changes. First-line drugs containing nonsteroidal anti-inflammatory drugs, paracetamol, and glucocorticoids, and joint replacement treatment mainly focus on relieving pain and mobility deficits and other symptoms instead of curing the disease directly [5–7]. For this reason, it is of great importance to find genetic biomarkers for OA to the benefit of its diagnosis and treatment.

Machine learning (ML) is an interdiscipline involving multiple subjects. As the core of artificial intelligence and data science, it focuses on how computers simulate or fulfill human learning behaviors so as to acquire new knowledge or skills and reorganize existing knowledge structures to constantly improve their performance. Given the big data era, ML is widely deployed in biomedical fields such as genomics, proteomics, microarrays, systems biology, evolution, and text mining [8–10]. Proposed by Tibshirani [11] in 1996, the LASSO algorithm (least absolute shrinkage and selection operator) obtains a refined model by constructing a penalty function. Its basic philosophy is to minimize the sum of square residuals under the constraint that the sum of the absolute values of regression coefficients is less than one constant, thus producing some regression coefficients that are strictly equal to zero and a model that has strong explanatory power. LASSO regression has the characteristic of screening variables and adjusting complexity while fitting the generalized linear models. Therefore, we can make models and predictions with it, irrespective of continuous, binary or multivariate discrete target factor variables. Support vector machine (SVM) is extensively adopted in pattern recognition, ML, and other fields. Support vector machine recursive feature elimination (SVM-RFE), a sequential backward selection algorithm based on SVM’s principle of maximum margin, trains the samples through the model, marks each feature, and rates the scores with the features of the lowest score being removed and the features left being used for training the model again, thereby performing iteration and finally selecting the desired features as supposed [12, 13]. Working with a mechanism integrating three parts, namely data input and SVM-RFE model construction, SVM classifier training and cross-validation, and error rate and accuracy rate calculation and mapping, SVM-RFE can better seek out feature genes of OA, consequently bettering its diagnosis and treatment. The feature genes selected through the LASSO regression model and SVM-REF model shall register higher reliability.

In recent years, immune infiltration has become more widely exercised in bioinformatics analysis, and there is evidence proving that cartilage cells in OA patients release specific antigens that trigger the activation of immune responses. There are a large number of immunocytes involved in OA, including innate immunity and acquired immunity, making anti-cytokines an ineffective treatment of the disease in question [14]. In this context, it is essential to elucidate the infiltration of immunocytes in the synovial membrane of OA patients and the genes involved in their regulation.

Based on the machine learning algorithm in bioinformatics, this paper aims to render a reference for revealing the complex pathogenesis of OA and developing more new markers for its diagnosis by exploring the feature genes related to its pathogenesis with R language tools and establishing the relationship between the genes and immunocyte infiltration.

## Data and methods

### Data download and integration

Three data sets GSE55235 and GSE55457 (respectively with 10 normal synovial tissues and 10 OA synovial tissues) and GSE98918 (with 12 normal synovial tissues and 12 OA synovial tissues) were downloaded from the GEO database [15, 16]. In addition, R language software was installed. Data integration and batch correction of the first two data sets were performed through the ‘sva’ package [17] in the R language to get integration results as training sets (20 OA synovial tissue samples and 20 normal synovial tissue samples). Meanwhile, GSE98918 [16] was collated to deliver a validation set.

### Screening of differential genes

The combined training set data were read in R language and divided into the OA group and the normal group (control group). The expression amounts of genes in each group were extracted, and the screening threshold value was set as |Log2FC|> 1.5, adjust. *p* value < 0.05, and a significant difference if both satisfied. Subsequently, the ‘limma’ package [18] was loaded to analyze the differences as per the above filter conditions and to output an analysis result, and the ‘sheetmap’ [19] and ‘ggplot2’ packages [20] were operated to draw heat map and volcano plot, respectively.

### Enrichment analysis of genes

GO, KEGG, and DO enrichment analyses on differential genes were conducted, respectively. The filter conditions for the enrichment analysis were defined as follows: *p* value < 0.05, q value < 0.05, and enrichment results with significance if both satisfied. The ‘org.Hs.eg.db’ package [21] was run for gene ID conversion, the ‘clusterProfiler’ package [22, 23] for enrichment analysis results, and the ‘enrichplot’ [24] and ‘ggplot2’ packages for results visualization and bubble and bar charts mapping. At last, GSEA enrichment analysis on all genes was carried out with the first five enrichment pathways being saved and plotted for visualization.

### Screening of feature genes

The ‘glmnet’ package [25] was subject to construct the LASSO regression model using differential genes, and the SVM-RFE model was built by loading ‘e1071’ [26], ‘caret’ [27], and ‘kernlab’ packages [28]. Based on the selected genes from the two models output and saved, the intersected genes were marked as feature genes, which were visualized by a Venn diagram drawn by the ‘venn’ package [29].

### Drawing of subject’s ROC curve

The ‘pROC’ package [30] was employed to plot the ROC curve of feature genes in the training sets. As AUC > 0.9, the genes could be deemed with higher accuracy in diagnosing disease.

### Verification of model results by the validation set

The expression of the selected feature genes in the validation set was analyzed using the ‘limma’ package, and the case of *p* value < 0.05 suggested that there was a difference between the expression of the genes in the OA group and those in the normal group. And the feature genes were plotted to obtain the ROC curve in the validation set, which was compared with the results obtained in the training set.

### Analysis of immunocyte infiltration

The abundance of various immunocyte types in the samples was computed through the ‘CIBERSORT’ algorithm [31]. First, the source code of CIBERSORT was created, and the expression quantity of marker genes of 22 immunocytes was prepared. Next, immunocyte infiltration analysis was performed for training set data, and *p* value < 0.05 was defined as the condition for filtering the analysis results being saved afterward. Then, the contents of immunocytes in samples were presented by a bar chart, and the correlation heat map was drawn using the ‘corrplot’ package [32]. Last, the ‘vioplot’ package [33] was utilized to draw a violin plot, which displays immunocytes showing the disparity in the OA group and normal group.

### Correlation analysis of feature genes and immunocytes

The ‘reshape2’ package [34] was adopted to sort out the gene expression data, obtain the expression quantity of feature genes, and circulate immunocytes with a correlation filtration condition set as *p* < 0.05. Furthermore, with a view to visualize the analysis results, scatter diagrams and lollipop diagrams for the correlation were plotted using the ‘ggpubr’ package [35] and ‘ggExtra’ package [36].

## Results

### Screening of differentially expressed genes

**Fig. 1:**
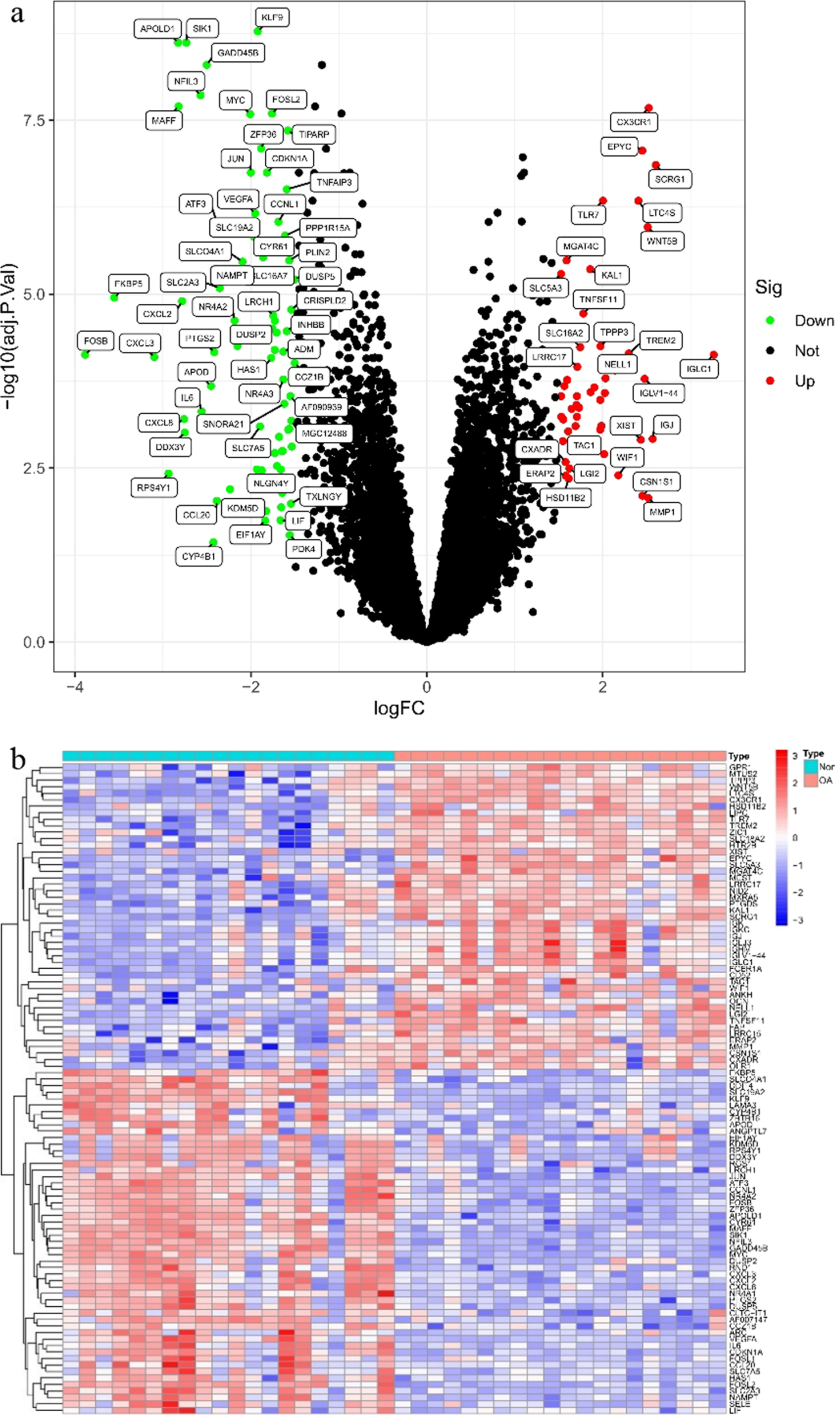
Screening of differentially expressed genes. **a** Volcano map of DEGs; red represents up-regulated differential genes, black represents no significant difference genes, and green represents down-regulated differential genes. **b** The thermal map of expression level of different genes in every synovial tissue sample, the redder the color, the higher the expression, the bluer the color, the lower the expression

We screened differential genes and found that 122 genes (including 48 up-regulated genes and 74 down-regulated genes) showed differences in expression quantity with a gap of over 2 times between the OA and normal groups (Fig. 1a,b).

### Gene enrichment results

#### Enrichment analysis results of differential genes

**Fig. 2:**
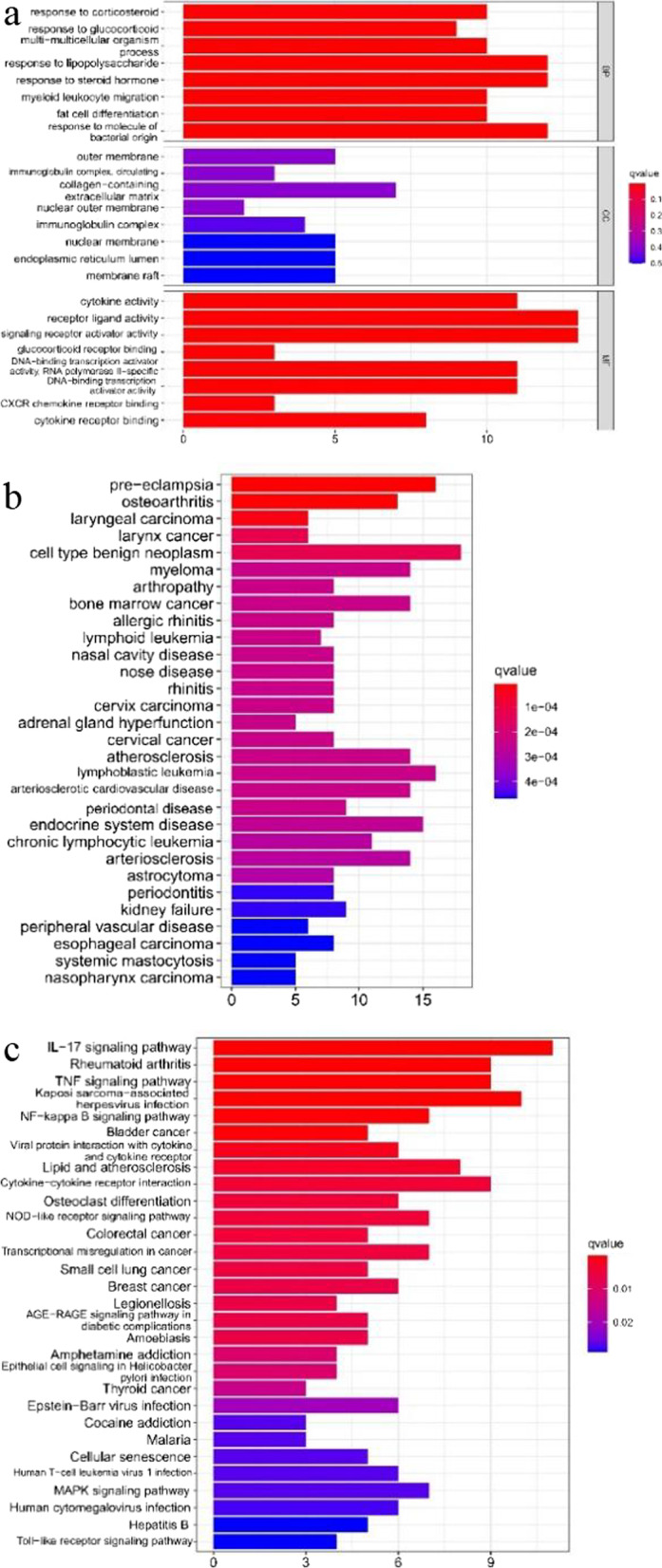
Gene ontology (GO), disease ontology (DO), and Kyoto Encyclopedia of Genes and Genomes (KEGG) enrichment analyses of DEGs. **a** GO enrichment analysis, where the horizontal axis represents the number of DEGs under the GO term. **b** DO enrichment analysis, where the horizontal axis represents the number of DEGs under the DO term. **c** KEGG enrichment analysis, where the horizontal axis represents the number of DEGs under the KEGG term

According to the gene ontology (GO) enrichment results, the main biological process (BP) involved by differential genes covers reactions to lipopolysaccharides, steroid hormones, and bacterial-derived molecules; cellular component (CC), in which the products of differential gene function, is mainly consisted of extracellular matrix (ECM), membrane rafts, membrane microdomain, etc.; and the molecular function (MF) of differential gene products encompasses receptor ligand activity, signal receptor activator activity, and cytokine activity and so on (Fig. 2a). It is derived from the KEGG pathway enrichment analysis that differential genes were largely involved in the signaling pathway of interleukin 17 (IL-17), followed by other pathways like herpesvirus infection associated with Kaposi sarcoma, rheumatoid arthritis, and tumor necrosis factor (TNF) (Fig. 2b). Moreover, the disease ontology (DO) analysis indicates that differential genes were concentrated in cell-type benign tumors, preeclampsia, lymphocytic leukemia, and osteoarthritis (Fig. 2c).

#### Analysis results of gene enrichment set

**Fig. 3:**
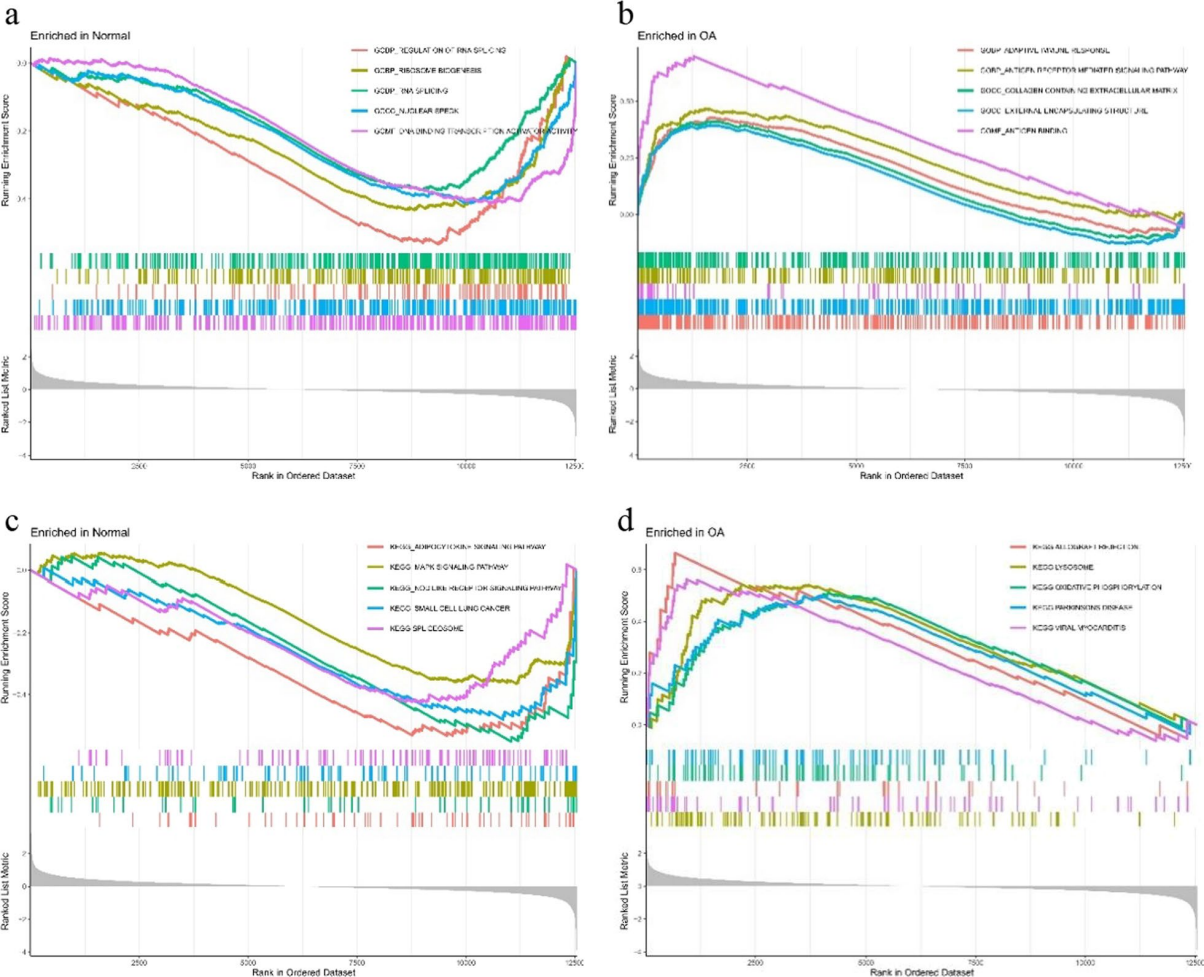
Gene GO and KEGG enrichment analysis of all normal genes and all OA genes. **a** GSEA-GO enrichment analysis on all normal genes, saved the top five enriched pathways. **b** GSEA-GO enrichment analysis on all OA genes, saved the top five enriched pathways. **c** GSEA-KEGG enrichment analysis on all normal genes, saved the top five enriched pathways. **d** GSEA-KEGG enrichment analysis on all OA genes, saved the top five enriched pathways

The GSEA-GO analysis found that the gene set of normal synovial tissues had DNA-binding transcription activator activity and that the product was functioning in nuclear speckles and was primarily engaged in RNA splicing control (Fig. 3a). The main products of the OA synovial tissue gene set played its role in binding antigen antibodies in the nuclear speckles (Fig. 3b).

The GSEA-KEGG analysis showed that the gene set of normal synovial tissues was mainly involved in signaling pathways such as adipocytokines, MAPK, and NOD receptors (Fig. 3c), whereas the gene set of OA synovial tissues substantially participated in signaling pathways of allogeneic rejection, lysosomal, and oxidative phosphorylation and other pathways (Fig. 3d).

#### Screening of feature genes

**Fig. 4:**
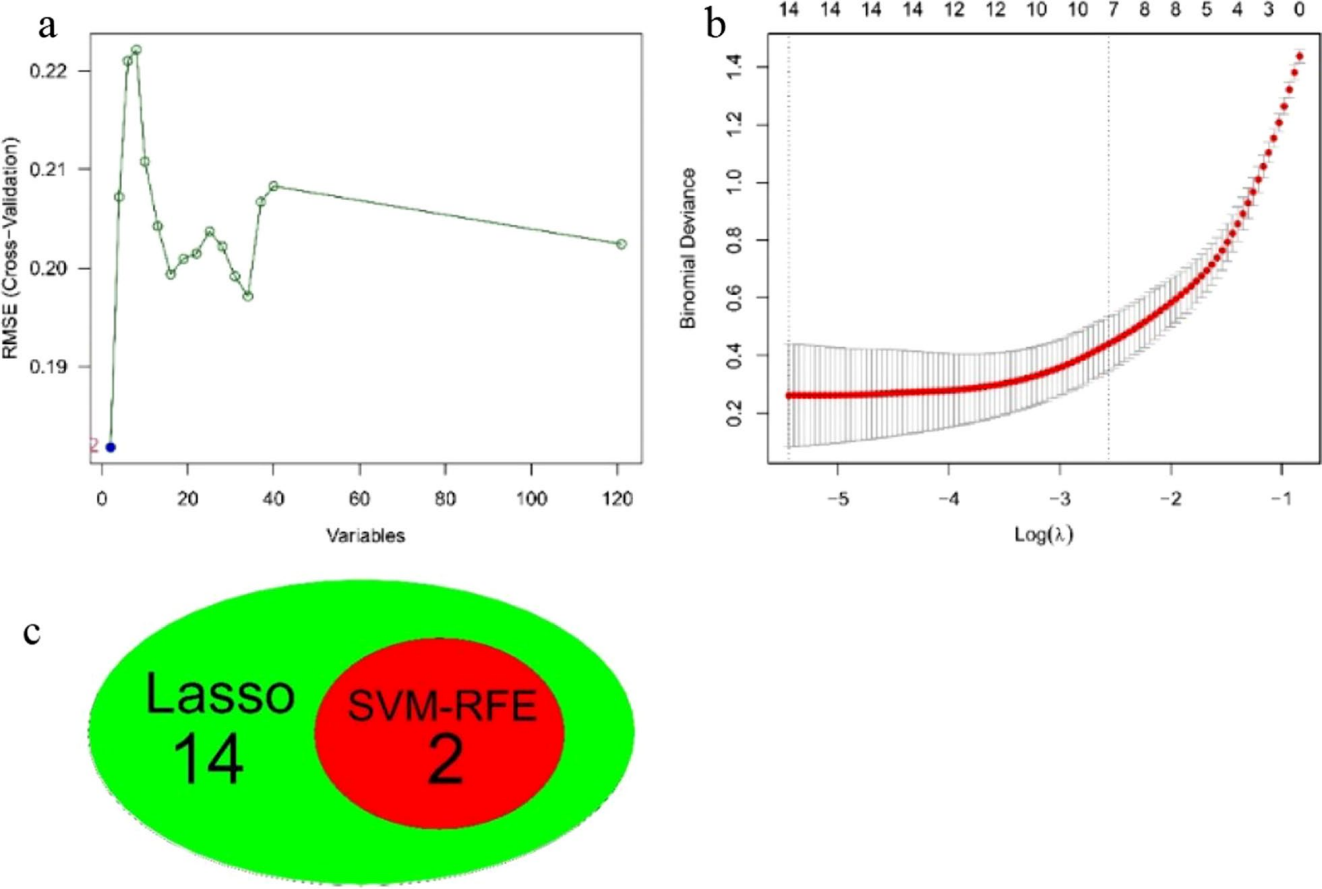
Screening of diagnostic markers. **a** Least absolute shrinkage and selection operator (LASSO) logistic regression algorithm to screen diagnostic markers. **b** Support vector machine–recursive feature elimination (SVM-RFE) algorithm to screen diagnostic markers. **c** Venn diagram shows the intersection of diagnostic markers obtained by the two algorithms

The LASSO regression model was established for the training sets, and the regression complexity was adjusted by the parameter λ, in which the greater the parameter, the greater the penalty for the linear model with more variables, hence obtaining a model with fewer variables. On such a basis, we screened out 14 feature genes with diagnostic significance (namely KLF9, APOLD1, TIPARP, EPYC, JUN, PPP1R15A, FKBP5, RND1, CCZ1B, ZIC1, MGC12488, TAC1, WIF1, and ERAP2) (Fig. 4a). To further construct the SVM-RFE model, two feature genes with diagnostic relevance (KLF9 and EPYC) were selected (Fig. 4b). Integrating the two regression models (Fig. 4c), we observed that KLF9 and EPYC can be used as feature genes of OA.

### Drawing of ROC curve

**Fig. 5:**
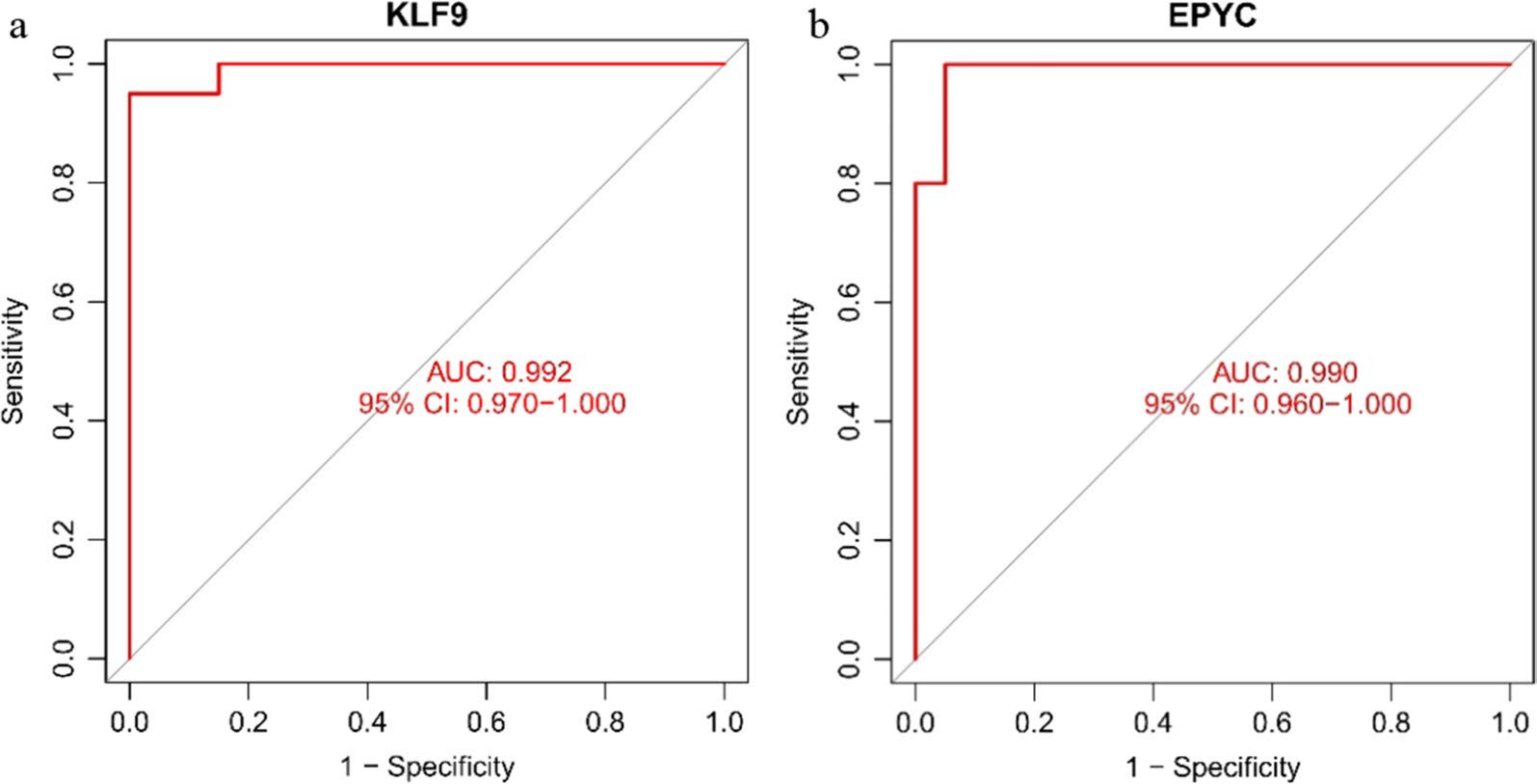
ROC curve of KLF9 (**a**) and EPYC (**b**) genes in the training and validation  set

Through plotting the ROC curves of KLF9 and EPYC, we noted that KLF9 (AUC = 0.992, CI = 0.97–1.00) and EPYC (AUC = 0.990, CI = 0.96–1.00) were relatively more sensitive in the diagnosis of OA (Fig. 5).

### Results of model validation

**Fig. 6:**
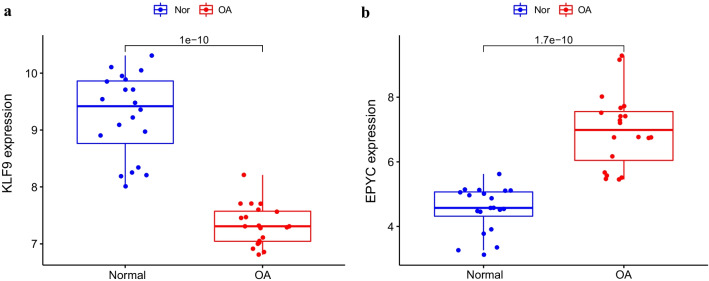
Box diagram of difference analysis of the expression levels of KLF9 (**a**) and EPYC (**b**) in the validation set. The blue marks represent the normal; the red marks represent the OA

The difference between the expression quantities of KLF9 and EPYC was analyzed in the validation set, suggesting that KLF9 was expressed at a low level in OA synovial tissue, while EPYC was expressed at a high level, which was statistically significant (*p* value < 0.05) (Fig. 6). Concurrently, observed from the ROC curves drawn for both genes in the validation set, the two genes were found to be sensitive in diagnosing OA (AUC > 0.9) (Fig. 5), which was consistent with the results of the training sets.

### Results of immune infiltration analysis

**Fig. 7:**
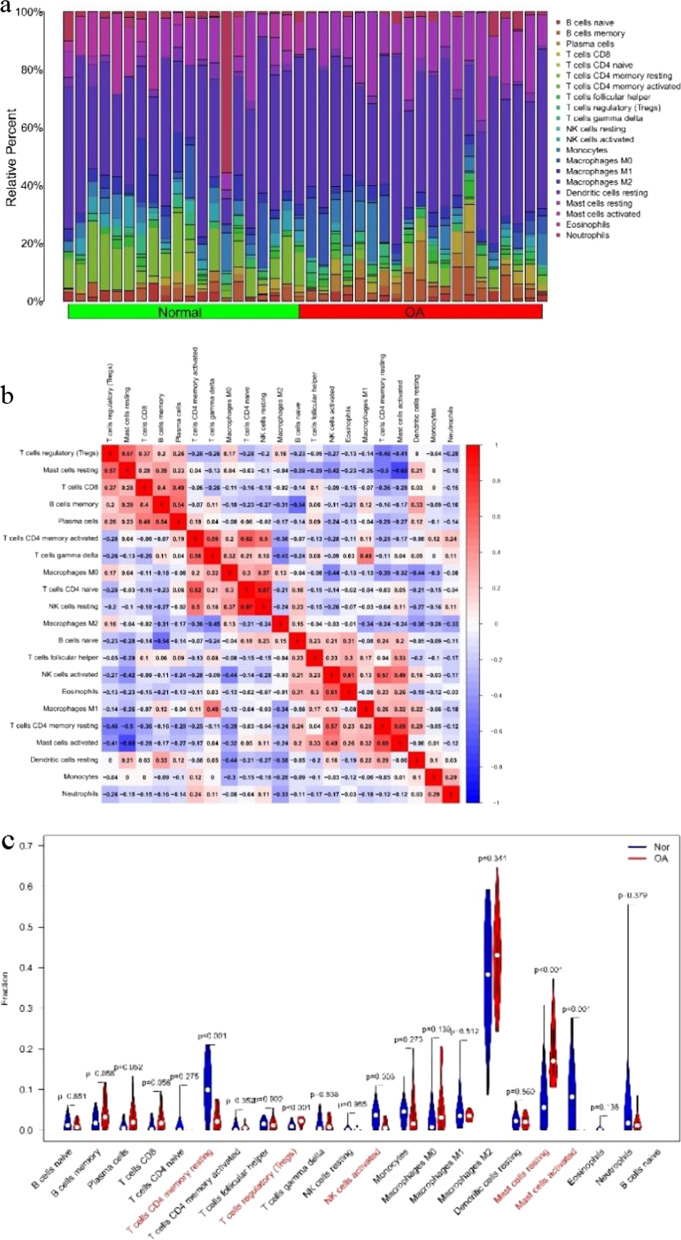
Evaluation and visualization of immune cell infiltration. **a** Content of different immune cells in each sample. **b** Correlation heat map of 22 types of immune cells. Red represents a positive correlation; blue represents a negative correlation. The darker the color, the stronger the correlation. **c** Violin diagram of the proportion of 22 types of immune cells. The red marks represent the difference in infiltration between the two groups of samples

The expressions of all immunocytes in each sample are shown in the bar chart (Fig. 7a). According to the analysis of the correlation between immunocytes, there was a positive correlation (correlation coefficient *R* > 0.5) between resting mast cells and regulatory T cells, plasma cells and memory B cells, γδT cells and activated CD4^+^ memory T cells, immature CD_4_^+^T cells and activated CD4^+^ memory T cells, resting NK cells and immature CD_4_^+^T cells, eosinophils and activated NK cells, and resting memory CD_4_^+^T cells and activated NK cells, as well as a negative correlation (correlation coefficient *R* <  − 0.5) between immature B cells and memory B cells, and between activated mast cells and resting mast cells.

The correlation between other immunocytes is exhibited in the heat map (Fig. 7b). There were five immunocytes expressed differently in OA and normal synovial tissues, of which resting memory CD_4_^+^T cells, activated NK cells, and activated mast cells were low expressed in OA synovium (*p* value < 0.01), while regulatory T cells and resting mast cells were highly expressed (Fig. 7c).

**Fig. 8:**
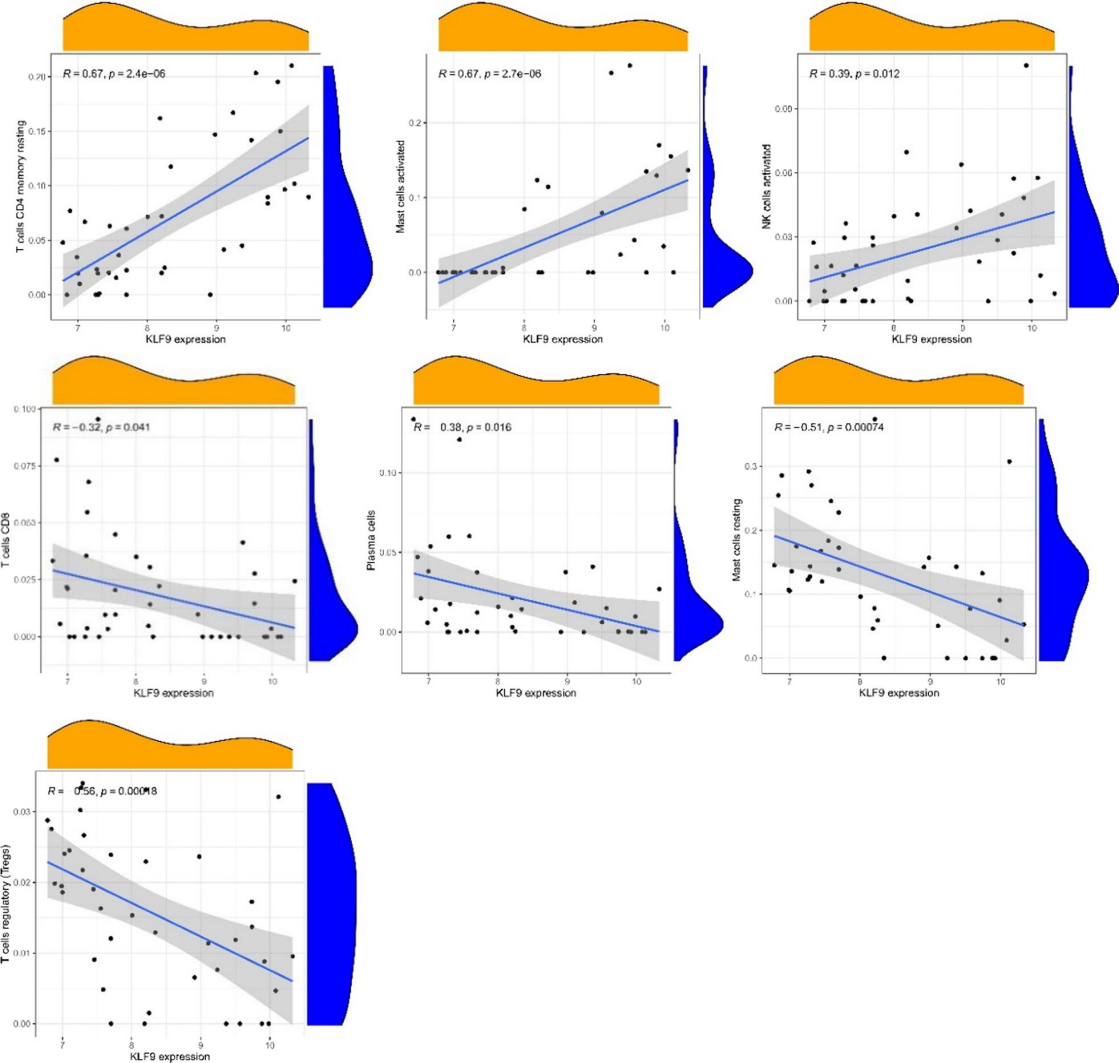
Correlation between KLF9 gene expression and different immune cells infiltrating

**Fig. 9:**
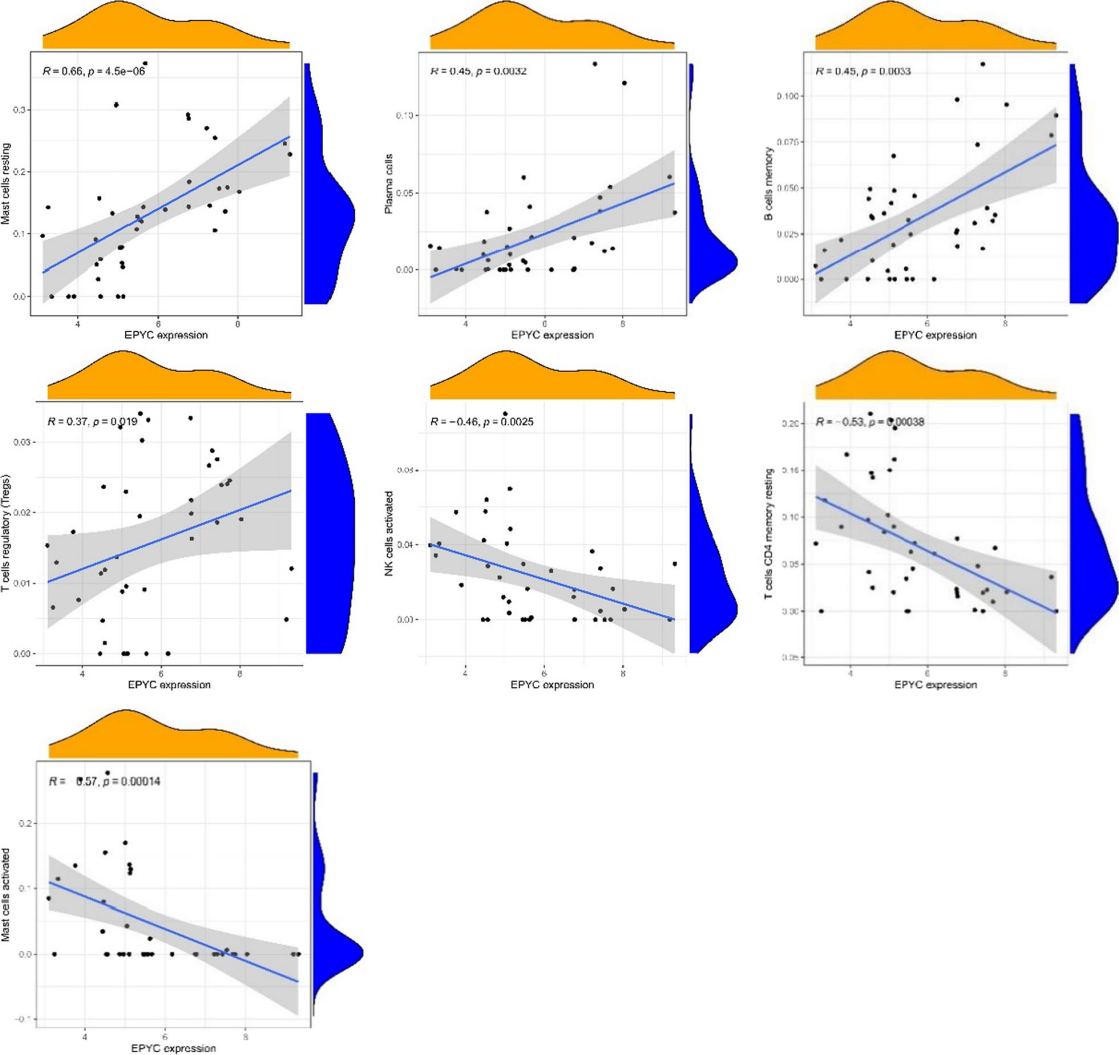
Correlation between EPYC gene expression and different immune cells infiltrating

**Fig. 10:**
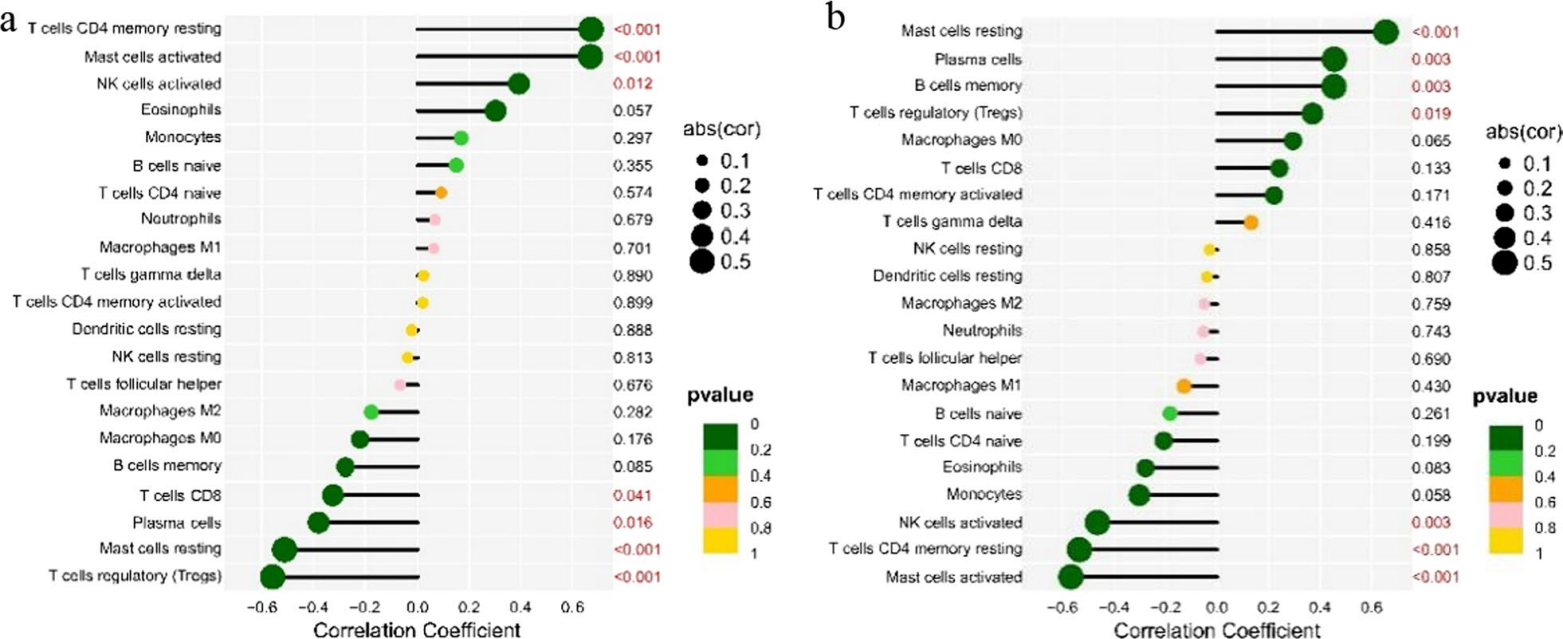
Correlation between KLF9, EPYC, and infiltrating immune cells. **a** Correlation between KLF9 and infiltrating immune cells. **b** Correlation between EPYC and infiltrating immune cells. The size of the dots represents the strength of the correlation between genes and immune cells; the larger the dots, the stronger the correlation, and the smaller the dots, the weaker the correlation. The color of the dots represents the *p* value; the greener the color, the lower the *p* value, and the yellower the color, the larger the *p* value. *p* value < 0.05 was considered statistically significant

From the co-expression correlation analysis of feature genes and immunocytes, it was revealed that KLF9 was positively correlated with the expressions of resting memory CD_4_^+^T cells (*R* = 0.67, *p* value < 0.01), activated mast cells (*R* = 0.67, *p* value < 0.01), and activated NK cells (*R* = 0.39, *p* value = 0.012), but negatively correlative with the expressions of CD_8_^+^T cells (*R* = − 0.32, *p* value = 0.041), plasma cells (*R* = − 0.38, *p* value = 0.016), resting mast cells (*R* = − 0.51, *p* value < 0.01), and regulatory T cells (*R* = − 0.56, *p* value < 0.01) (Figs. 8, 10a). EPYC was positively associated with the expressions of resting mast cells (*R* = 0.66, *p* value < 0.01), plasma cells (*R* = 0.45, *p* value < 0.01), memory B cells (*R* = 0.45, *p* value = 0.01) and regulatory T cells (*R* = 0.37, *p* value = 0.019), while it was negatively correlated with the expressions of activated NK cells (*R* =  − 0.46, *p* value < 0.01), resting CD_4_^+^T memory T cells (*R* =  − 0.53, *p* value < 0.01), and activated mast cells (*R* =  − 0.57, *p* value < 0.01) (Figs. 9, 10b).

## Discussion

OA with the most common joint disease incidence among the elderly causes heavy public health burdens but cannot be fully cured. Since articular cartilage with the function of reducing friction is the most degenerative part once OA occurs, reconstructing the complete articular cartilage is expected to be a radical cure for OA in replacement of the current prevalent treatment of articular replacement.

There are two main types of enrichment analysis methods: general enrichment based on the number of differential genes and functional GSEA based on gene ranking. The former focuses on genes with large differences, without considering the genes with fewer differential expressions but significant biological significance in the pathopoiesis of OA, which can be compensated by the latter. Lipopolysaccharides have many mechanisms in the development of OA and are more significantly associated with the disease in obese populations. It can initiate innate immune responses with toll-like receptor 4, leading to systemic inflammatory responses and damage to joint structures [37], which is in accord with our GO analysis results. IL-17 plays a key role in the pathogenesis of OA and is closely related to pain. Up-regulation of many gene products involved in cell activation, including human macrophages, can also increase the production of NO in chondrocytes and induce apoptosis in the progression of the disease discussed here [38, 39]. Chondrocyte apoptosis is one of the principal causes of OA, and the death of chondrocytes in OA cartilage is primarily attributed to autophagy defect, mitochondrial dysfunction and increased oxidative stress. The lysosome is highly related to autophagy, and the loss of its function leads to the accumulation of dysfunctional mitochondria and then participates in the formation of OA [40], which coincides with the findings of our GSEA enrichment analysis and also proves the accuracy of our study. Pursuant to our DO enrichment analysis, a high concentration of genes with differential expressions and cell-type benign tumors may be of limited clinical significance. However, according to the literature, studies have shown that in OA there are 12q13-15 chromosome mutations pertain to benign mesenchymal tumors [41], which may offer a new perspective on how OA progresses.

Oxidative stress and reactive oxygen species (ROS) have been demonstrated to be strongly associated with the occurrence of OA. When chondrocytes, synoviocytes, and osteoblasts are subject to constant external mechanical stress, they can produce excessive pro-inflammatory mediators to break the pro-oxidative/antioxidant balance, hence the degradation of ECM [42]. As a member of the KLFs family, KLF9 (Kruppel-like factor 9) plays a significant role in oxidative stress responses. Studies have suggested that Nrf2 stimulates the expression of KLF9 and inhibits the expression of several important antioxidant enzymes such as thioredoxin reductase 2, resulting in the increase in KLF9-dependent ROS and ultimately cartilage degradation [43]. The KEGG pathway enrichment analysis showed that differentially expressed genes in OA were mainly involved in the IL-17 signaling pathway, and IL-17 could also impel the process of oxidative stress, so further investigation is needed to see if there is a potential link between OA and IL-17.

Dermatan sulfate proteoglycan (Epiphycan, EPYC), a protein-coding gene and a member of the small leucine-rich proteoglycans (SLRP) family, consists of seven exons and regulates fibrillogenesis by interacting with collagenous fibrils and other ECM proteins. EPYC is involved in cartilage formation in normal synovial tissues, and OA occurs with age in mice with EPYC knockout [44, 45]. In this study, EPYC was overexpressed in OA, possibly because the destructed articular cartilage led to the increase in EPYC production by chondrocytes in an attempt to repair the damaged ECM. Considering that EPYC is a member of the SLRPS family, whose effects on cartilage and the pathogenesis of OA are various and complex, including changes in extracellular collagen networks and TGF-b signaling pathways, the regulation mechanism of EPYC in OA needs to be further elucidated. Moreover, NSAIDS drugs, as the first-line treatment for OA, have been proven effective in curbing the expression of EPYC in prostate cancer cells [46], but their effects on EPYC gene expression in OA articular chondrocytes should be further explored.

CIBERSORT score is widely and accurately used in gene expression profiling to quantify immune cell fraction. The infiltration of immunocytes in OA synovial tissues has been accepted by many scholars. CD_4_^+^T cells, mast cells, and macrophages play an essential role in synovitis. The pathogenesis of activated IgE-dependent mast cells and mast cell-mediated tryptases in OA has been demonstrated [47], but there is no difference in the expression of mast cells themselves in OA synovial tissues. In this study, the immune infiltration analysis herein showed that resting mast cells were highly expressed in OA synovium, while activated mast cells had low expression. We speculate that it may be ascribed to the fact that mast cells were not directly involved in the pathogenic process of OA but indirectly caused the disease by mediating other proteases or histamine, and others. Nevertheless, the specific mechanism of mast cells in OA needs further research. Regulatory T cells (Tregs) play an important immunomodulatory part in many inflammatory and autoimmune diseases and can inhibit osteoclasts and helper T cells to protect local articular cartilage from destruction [48–50]. Our experimental results signified that Tregs were infiltrated the OA synovial membrane, probably owing to the fact that the destruction of synovial tissues may result in the reactive proliferation of Tregs, thereby inhibiting local inflammatory responses, which requires further verification by experiments.

This is not the first time that machine learning algorithms have been applied to sort out genes for osteoarthritis. From past experiments, we believe that excessive differential genes due to low difference threshold have affected the accuracy of enrichment analysis and machine learning algorithms. Under such a circumstance, we tripled the threshold for differential analysis, i.e., Log2FC was set to 1.5, which was supposed to be more accurate and relevant.

## Conclusion

The analysis of the OA gene expression chip showed that silencing of KLF9 and overexpression of EPYC were highly relevant to the occurrence of OA, and the two genes could be taken as diagnostic feature genes of OA based on machine learning algorithms. In OA synovium, resting memory CD_4_^+^T cells, activated NK cells, and activated mast cells were suppressed, while regulatory T cells and resting mast cells were overexpressed. KLF9 was positively correlated with CD_4_^+^T cells, activated NK cells, and activated mast cells, but negatively correlated with CD_8_^+^T cells, plasma cells, resting mast cells, and regulatory T cells. EPYC showed positive correlations with plasma cells, resting mast cells and regulatory T cells, and negative ones with resting memory CD_8_^+^T cells and activated mast cells.

In conclusion, we processed the chip expression data by computer, used machine learning algorithms to find out the diagnostic feature genes of OA, and explored their relationships with immunocytes, so as to provide a reference for the early diagnosis and treatment of OA.

## Data Availability

All data generated or analyzed during this study are available from the author.
